# Developmental Biology: An Introduction and Invitation

**DOI:** 10.3390/jdb8030011

**Published:** 2020-06-28

**Authors:** Simon J. Conway

**Affiliations:** Herman B. Wells Center for Pediatric Research, Indiana University School of Medicine, Indianapolis, IN 46202, USA; siconway@iu.edu

## 1. Background

Developmental biology is arguably the most exciting and evolving field of study within the biological sciences. It encompasses experimental examination of the development of multicellular organisms’ growth, differentiation and remodeling to give rise to the adult form, using molecular, cellular, tissue, organ and whole organism methodology. Understanding how embryos form, grow and shape remains fundamental to developmental biologists but now also includes the exciting fields of growth and differentiation of stem cells in adults, the biology of organ regeneration and evolutionary developmental mechanisms. Moreover, the diverse range of different animal model systems used by developmental biologists (*Drosophila, Caenorhabditis elegans, Xenopus*, Zebrafish, chick, mouse, embryonic and induced pluripotent stem cells et al.) makes for a better understanding of systems biology and the scale of organogenesis, pattern formation and regeneration.

Embryonic stem cells encode a nearly unlimited self-renewal capacity and retain the developmental potential to differentiate into most cell types within an organism and represent an inherent model of embryonic development and tissue regeneration. Moreover, most adult organs maintain a stem cell population sharing important parallels with embryonic progenitors, which includes the ability to both self-renew and to differentiate into the full range of the specialized cell types corresponding to the different organs in which they reside. The establishment of pluripotential stem cells from mouse [1] and human [2] tissues has resulted in multiple breakthroughs in our understanding lineage commitment as well as cell therapies in regenerative medicine as stem cells can be transplanted or manipulated in vivo to restore missing cells. Similarly, the successful reprogramming of somatic cells into induced pluripotent stem cells [3] has expanded understanding of human development and organoid research, based on an unlimited, renewable source of tissue progenitor cells. Studying regeneration and reemergence of embryonic organizers in animals where and when it occurs is inherently interesting and a challenging research topic within developmental biology [4]. More recently, light is being shed on the different signaling pathways in development that indicate how cells communicate to coordinate building an organism from a single cell [5]. Most biomedical research and regenerative medicine have also been shaped and the groundwork laid via developmental biology. For instance, the bulk of today’s assisted reproductive techniques originated in work that had been initiated in animal developmental biology [6]. Similarly, our understanding of adult heart disease has been greatly advanced via a heightened appreciation of murine cardiovascular developmental biology, particularly in the case of pathological hypertrophy wherein the phenotype was found to resemble those observed during in utero cardiac development [7]. Significantly, the reactivation of a “fetal gene program” is believed to play a causative role in adverse cardiac remodeling and the pathogenesis of heart failure in patients [8]. Finally, although Hedgehog signaling was first discovered as a driver of *Drosophila* segmentation, it is now known as key regulator of embryonic development and tissue homeostasis and its dysfunction underlies a variety of human congenital anomalies and diseases. Moreover, Hedgehog signaling is presently recognized as a major target for cancer therapy as well as a mediator of directed stem cell differentiation [9]. 

The above are only some of the many examples of the importance of developmental biology research for biomedical science as a whole, and they will surely not be the last, as development still has a long way to go to understand the entire lifespan of organisms until their demise. As recently elegantly stated, developmental biologists have “no more ‘solved’ the embryo than we have ‘solved’ cancer” [10]. Therefore, there is undoubtably a lot on interesting and innovative developmental biology still to be done and still to be published.

## 2. A Wide Spectrum of Topics Will Be Covered in the EBMs Special Issue

This Special Issue focuses on development mechanisms and genetics, cell differentiation, embryonal development, tissue/organism growth, and the regeneration of the organisms. It involves many biological fields, such as molecular biology, genetics, physiology, cell biology, anatomy, embryology, cancer research, regenerative medicine, neurobiology, immunology, and evolutionary biology. The aim of the *Journal of Developmental Biology (JDB)* is to encourage researchers to effortlessly publish their new findings or concepts rapidly in an open-access medium, overseen by their peers.

It is dedicated to the many recent advances in the research area of developmental biology of exclusive papers of the Editorial Board Members (EBMs) of the *JDB*, or those recommended and invited by the Editorial Board Members and the Editor-in-Chief. Our new Special Issue can be also viewed as a way of introducing the *JDB’s* EBMs to top-notch researchers, so they will consider our journal as an attractive open-access publishing platform for displaying their scientific data.
